# The value of genetic testing in pediatric and adult ophthalmology

**DOI:** 10.1515/medgen-2024-2059

**Published:** 2025-02-12

**Authors:** Ulrich Kellner, Simone Kellner, Silke Weinitz, Ghazaleh Farmand

**Affiliations:** Center for Rare Retinal Diseases AugenZentrum Siegburg, MVZ Ophthalmological Diagnostic and Therapy Centre Siegburg GmbH Europaplatz 3 53721 Siegburg Germany; Center for Rare Retinal Diseases Eye Center Siegburg, MVZ Ophthalmological Diagnostic and Therapy Centre Siegburg GmbH Europaplatz 3 53721 Siegburg Germany; Center for Rare Retinal Diseases Eye Center Siegburg, MVZ Ophthalmological Diagnostic and Therapy Centre Siegburg GmbH Europaplatz 3 53721 Siegburg Germany; Center for Rare Retinal Diseases Eye Center Siegburg, MVZ Ophthalmological Diagnostic and Therapy Centre Siegburg GmbH Europaplatz 3 53721 Siegburg Germany

**Keywords:** inherited retinal dystrophies, inherited optic neuropathies, genetic testing, retinal imaging, patient relevant outcome

## Abstract

Inherited retinal dystrophies and optic neuropathies (IRD) are the most frequent cause for vision loss in the working age. The huge variability of phenotypes and initial clinical presentation frequently delay the ophthalmologic diagnosis. The most frequent phenotypes are retinitis pigmentosa, macular dystrophies, cone-rod dystrophies and syndromes associated with IRDs. Causative gene variants have been identified in more than 300 genes, with a frequency variation between different ethnicities. In this series of 1 914 patients seen in Germany between 1995 and 2024, in 47.4 % of families the genetic background could be solved. Even with a common genotype, the phenotype can be variable. Genetic diagnostic testing is important for the correct diagnosis, for patient selection for current or future therapies, but also from the patient perspective.

## Introduction

**Figure 1:**
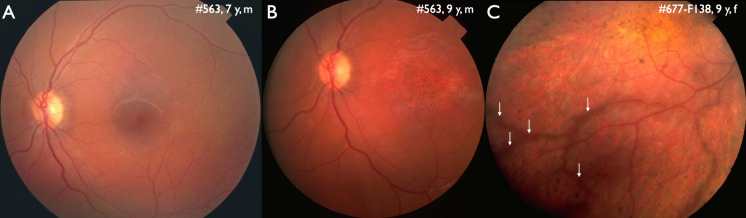
Fundus photography. A,B. A male patient affected by autosomal recessive Stargardt disease (*ABCA4*: c.768G>T, c.2564G>A). A. At the age of 7 years temporal optic disc pallor is the earliest sign of the disease. B. 2 years later, pigmentary alterations have developed in the macula, the difference in color is due to different camera systems. C. 9 years old female patient affected by retinitis pigmentosa with preserved para-arterial pigment epithelium (white arrows) in an otherwise dystrophic retina. This patient presented with an early onset IRD associated with disease-causing variants the *CRB1* gene (c.2234C>T, c.2843G>A) and autosomal recessive inheritance.

Multiple ophthalmologic disorders are associated with alterations in the genetic profile. Some disorders are associated with genetic predisposition (e. g. age-related macular degeneration, diabetic retinopathy), and some tumors are associated with germline or somatic mutations (e. g. retinoblastoma, uveal melanoma, Hippel-Lindau disease). There are multiple monogenic disorders affecting various cells and tissues of the eye. Inherited retinal dystrophies (IRD) including optic neuropathies (ON) are the largest group of those and will be covered in this review. From a clinical point of view a differentiation between IRD and ON is of limited value. The majority of IRDs affecting the photoreceptor/retinal pigment epithelium complex present with alterations of the optic disc. A pale temporal optic disc can be the first sign of macular dystrophies on ophthalmoscopy and can be misinterpreted as ON (Fig. 1A,B). In those cases, genetic testing would be unsuccessful when limited to ON. In addition, separate alterations of the macula and the optic nerve in the same eye are associated with several genes (e. g. *SSBP1*, *BRAT1*, *CASK*).

## Clinical presentation and diagnostic approach

Based on the carrier frequency of pathogenic gene variants [1] and population based studies [2] it can be estimated that up to 80 000 persons are affected with IRD in Germany. Though this does not seem infrequent, the heterogeneous group of IRD is split up in a huge variety of phenotypes. The most frequent group of disorders is retinitis pigmentosa (46 %) followed by macular dystrophies (16 %) and cone-rod dystrophies (13 %) [3]. At least 80 syndromes are associated with IRDs with a total of 9 % of all IRDs [3,4].

The age of onset varies between presentation at birth (Leber congenital amaurosis) and first symptoms beyond 60 years of age in some patients with retinitis pigmentosa or Best disease. Most patients develop symptoms within the first three decades of life. The onset of symptoms is often not immediately apparent to patients and in most IRDs slowly progressive over many years. However, in patients with Leber hereditary optic neuropathy (LHON) bilateral severe visual loss develops within weeks, and in macular dystrophies, e. g. Stargardt disease, loss of reading ability may develop within a short period of few months.

Unfortunately, the clinical diagnosis is often delayed, sometimes for years [5,6]. This is due to two major obstacles for an early diagnosis. First, initially the patient’s symptoms are often unspecific and overlap with a variety of non-genetic ocular disorders. Second, at first presentation retinal alterations may be either completely absent or unspecific, while characteristic textbook changes appear much later in the course of the disease. Therefore, it is recommended, that in all patients with otherwise unexplained visual loss, an IRD should be suspected, and the relevant diagnostic process should be initiated.

The diagnostic process has markedly changed in the last decade. Non-invasive high-resolution retinal imaging (NIRI) with optical coherence tomography (OCT), fundus autofluorescence (FAF), near-infrared autofluorescence (NIA) and OCT angiography (OCTA) provide a detailed examination of the retina, retinal pigment epithelium and choroid [7]. Especially OCT, FAF and NIA have markedly improved the early and differential diagnosis of IRDs. OCT and FAF are widely available, allow easy follow-up and an examination even in infants. Several biomarkers have been identified, which are indicative of IRDs, though not associated with specific genes. However, as these are rare diseases, the knowledge especially of mostly subtle, early alterations is not widespread in ophthalmologists. Therefore, it is recommended that patients with suspected IRD are referred to specialized centers, most of them are connected via the DRN-Eye network (drn-eye.de). Due to NIRI, electrophysiologic methods no longer play a major role in the diagnostic process. These are less frequently available, more time consuming, require more patient cooperation and the results show a higher variability between visits [7]. This might differ in special circumstances e. g. for selecting patients for certain gene therapies [8].

The recently revised ophthalmologic guideline for the diagnosis of IRDs [9] recommends that ophthalmologists initiate genetic testing and recommend genetic counselling. However, currently genetic testing is mostly initiated in specialized centers.

## Genetic background

The genetic landscape of IRDs is even more heterogeneous than the clinical presentation. More than 300 genes have been documented to be associated with one or more clinical phenotypes [10,11]. In some of these genes causative variations have only been identified in one or few families. Within these IRD-associated genes, often hundreds of pathogenic or likely pathogenic variants have been found.

The frequency of genes associated with IRDs differs between ethnicities. The most frequent genes associated with IRDs in Germany are *ABCA4* (19.2 % of solved or likely solved families), *USH2A* (5.9 %) and *RPGR* (5.5 %) in a recent study [12] and *ABCA4* (20 %), *PRPH2* (8 %) and *USH2A* and *BEST1* (both 6 %) in the present study. Similarly, in Great Britain the distribution is *ABCA4* (21 %), *USH2A* (9 %), *RPGR* and *PRPH2* (both 5 %) [13]. In contrast, in Israel the most frequent genes are *ABCA4* (8 %), *EYS* (7 %) and *USH2A* (6 %) [10] and in Japan the most frequent genes are *EYS* (19 %), *RP1L1* (6 %) and *RPGR* (5 %) [14].

## Clinical value of genetic diagnostics

Basically, there is only one IRD phenotype that can be associated with a specific gene: retinitis pigmentosa with preserved para-arterial pigment epithelium (RP12) is associated with the *CRB1* gene (Fig. 1C) [15]. There are two other disorders with specific electroretinographic findings associated with a specific gene: Enhanced S cone syndrome (*NR2E3*) and cone dystrophy with supernormal b-waves (*KCNV2*). All other phenotypes, identified either with funduscopy, NIRI or electrophysiology are associated with more than one gene.

In the age of gene therapy, with one certified therapy for *RPE65* associated IRDs available to patients [16] – most likely all identified patients in Germany have been offered or underwent therapy – and a multitude of clinical trials ongoing, the identification of causative genetic alterations is important to identify patients for possible treatment.

The rate of solved and likely solved cases is dependent on the genetic techniques used and the clinical disorder, and ranges between 35 to 95 % [3]. The patients reported here were examined in centers focused on IRDs in Germany (Berlin, Siegburg) between 1995 and 2024 by one of the authors (U. K.). In this series of 1 914 patients from 1 615 families who underwent genetic testing ranging from single gene evaluation to whole genome sequencing, 765 families (47.4 %) were defined as solved or likely solved (one monoallelic pathogenic or likely pathogenic variant in autosomal dominant, X-linked and mitochondrial IRDs, two biallelic pathogenic or likely pathogenic variants in autosomal recessive IRDs). In the last decade with more detailed testing, the rate of solved or likely solved cases increased (333/613 families, 54.3 %). Detailed data on clinical diagnosis, gene causes (genes and variants) on the huge majority of these patients have been published recently [17].

In our series of solved and likely solved cases, disease-associated variants were detected in 101 different genes. Only 18 genes were associated with at least 10 families (Table 1). The distribution in our series was different compared to both recent series from Germany of Weisschuh et al [3,12]. This reflects the heterogeneity of the respective patient populations. There was a higher percentage of patient with Asian ethnicity in that group compared to ours [3].

Though genetic testing is important for verifying the diagnosis, the ability to predict the future course of the disease is limited. There have been multiple attempts to establish genotype-phenotype correlations, which have been successful only to a very limited degree. Variants in the *ABCA4* gene can be associated with autosomal recessive IRDs ranging from macular dystrophy (i. e., Stargardt disease) limited to the posterior pole to severe cone-rod dystrophy and rarely retinitis pigmentosa, both of the latter involving all of the retina. Even within the group of *ABCA4*-associated macular dystrophies there is a subgroup with predominantly perifoveal atrophy, in whom the preserved foveal center is associated with a maintained reading ability for many years. In large series of *ABCA4* associated IRDs, the clinical phenotype was more predictive for the future course of the IRD in an individual patient compared to the genetic findings due to the high variability of phenotypes [18].

**Table 1:** Gene distribution in solved/likely solved families

***Gene***	***Inheritance*** ***present study***	***Families n=765***	***Frequency ranking***	
			***[3]***	[12]
*ABCA4*	ar	150	1	1
*PRPH2*	ad	62	6	6
*USH2A*	ar	49	2	2
*BEST1*	ad	49	12	8
*RS1*	X	44		10
*RPGR*	X	36	3	3
*OPA1*	ad	36		5
*RHO*	ad	28	4	11
*CHM*	X	23	7	8
Mitochondrial DNA	mito	19		
*PRPF31*	ad	14	5	4
*EYS*	ar	14	8	7
*RP1L1*	ad	12	11	
*OPN1LW*	X	12		14
*CNGA3*	ar	10		12
*CNGB3*	ar	10	9	11
*CRB1*	ar	10	10	12
*NR2E3*	ar	10	14	15
*GUCY2D*	ad	8		

ad: autosomal dominant, ar: autosomal recessive, mito: mitochondrial, X: X-linked

**Figure 2:**
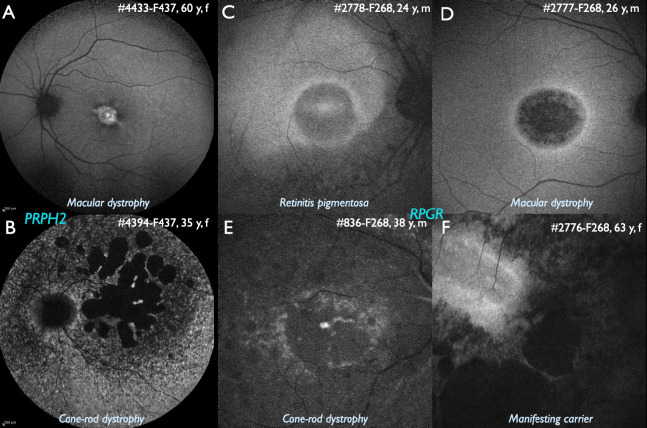
Variability of the phenotype in *PRPH2* and *RPGR* associated IRD. A,B Family 437, autosomal dominant IRD associated with a pathogenic variant in *PRPH2* (c.715C>T): A. Wide-field fundus autofluorescence (FAF) in the 60 years old mother displaying a central vitelliform lesion (i. e., adult vitelliform macular dystrophy) B. Wide-field FAF in the 35 years old daughter shows peripapillary preserved FAF, large areas of absent FAF, indicating loss of the retinal pigment epithelium, and multiple patchy irregularities throughout the remaining retina. C,D,E,F: Family F268, a kinship of three affected brothers and their affected carrier mother due to X-linked IRD associated with a pathogenic variant in the *RPGR* gene (ORF15 c.2740G>T). Family members were examined at the same date at the age indicated. C. FAF in the youngest brother (24 years old) shows a pericentral ring of increased intensity and mid-peripheral loss of intensity indicating retinitis pigmentosa. D. FAF in the middle brother (26 years old) shows a pericentral ring of increased intensity and central intensity loss indicating macular dystrophy. E. FAF in the oldest brother (38 years old) shows some patchy areas of preserved intensity indicating cone-rod dystrophy. F. FAF in the 63 years old mother shows irregular areas of preserved intensity which cannot be differentiated in either retinitis pigmentosa or cone-rod dystrophy.

Especially variants in *PRPH2* (autosomal dominant) and *RPGR* (X-linked) have been frequently reported with a huge phenotypic variability even in the same family, including macular dystrophy, cone-rod dystrophy and retinitis pigmentosa [19,20] (Fig. 2). Therefore, counselling of possible affected family members should be performed very cautiously. Different phenotypes have also been reported in several other genes [11,21].

In addition, in a phenotype that has been clearly associated with one genetic profile, e. g. LHON with causative variants in the mitochondrial DNA, the genetic spectrum has unexpectedly broadened to include an autosomal recessive LHON associated with biallelic variants in the *DNAJC30* gene [22] rising the question whether treatment with Idebenone [23] is helpful for both genetic backgrounds.

**Figure 3:**
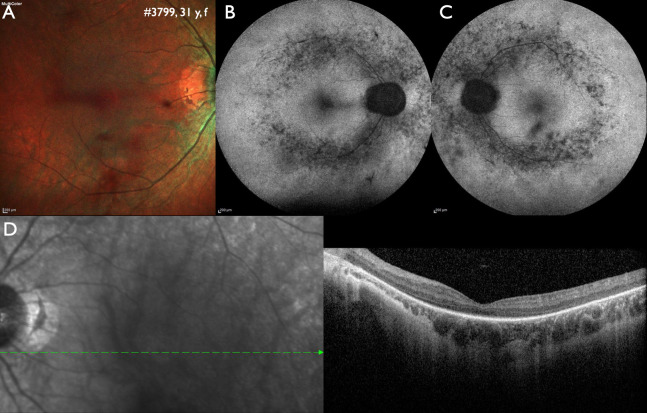
Correlating genotype and phenotype when disease-causing variants in more than one gene may be involved. Variants in four genes were identified in this 31 years old female patient. A homozygous pathogenic variant in *IMPG2* (c.1578_1581del) is most likely causative for the phenotype of autosomal recessive retinitis pigmentosa. Secondly, a likely pathogenic variant in *RPGR* (c.1094C>T) could indicate a manifesting carrier of X-linked retinitis pigmentosa, however, characteristic alterations of X-linked retinitis pigmentosa are missing in the fundus autofluorescence images. Thirdly, a likely pathogenic variant in *CRX* (c.425A>G) could be associated with early onset Leber congenital amaurosis, which did not fit to the patient history, or autosomal dominant cone-rod dystrophy, which did not fit to the phenotype either. Lastly, a single likely pathogenic variant in *USH2A* (c.4861A>G) was found, but can be excluded as causative as variants in *USH2A* are inherited as an autosomal recessive trait. A: Fundus image of the right eye showing midperipheral atrophy. B and C: Wide-field fundus autofluorescence of the right and left eye shows patchy reduced intensity along the vascular arcades, the area with the highest rod photoreceptor density. D. Optical coherence tomography shows a reduction of the outer retinal layers in the center and absence of outer retinal layers towards the periphery.

More than one gene with pathogenic or likely pathogenic variants was found in 36/765 (4.7 %) solved or likely solved families. If no family members are available for further confirmation, a comparison of phenotype and genotype may help to indicate the most likely causative gene (Fig. 3), but also double genotype cases exist.

In addition, in 94/765 families (12.3 %) one or more genes with variants of uncertain significance were identified. It remains to be defined, whether and to which degree these variants may influence the phenotype and the clinical course of the patients.

**Figure 4:**
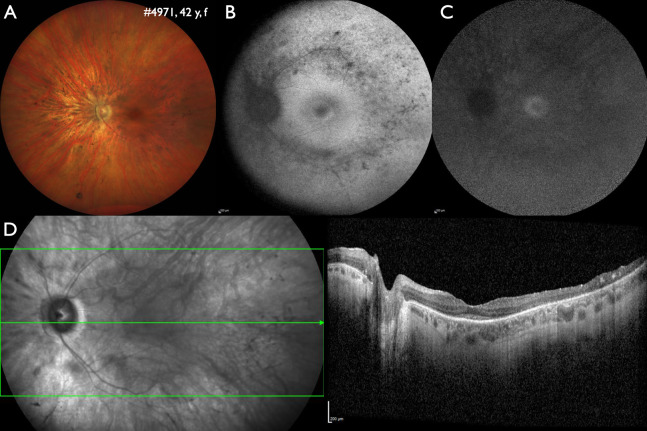
Three genes, but no clarification: In this 42 years old female patient with isolated retinitis pigmentosa two variants of uncertain significance were identified in both *ADGRV1* (c.4000G>C, c.14320C>T) and *SCLT1* (c.618_621del, c.1597A>G). Disease-causing variants in *ADGRV1* are associated with autosomal recessive retinitis pigmentosa and hearing loss (Usher syndrome), no hearing problems were noted by the patient, but this was not tested. Disease-causing variants in *SCLT1* have been rarely associated with autosomal recessive syndromic (under discussion: non-syndromic) IRDs. In addition, a variant of uncertain significance was identified in *IMPG1* (c.919A>G). Disease-causing variants in *IMPG1* are associated with retinitis pigmentosa or vitelliform macular dystrophy with either autosomal recessive or dominant inheritance. A: Wide-field fundus image of the left eye showing midperipheral atrophy. B: Wide-field fundus autofluorescence of the right eye shows patchy reduced intensity along the vascular arcades and a paracentral ring of increased intensity, marking the outer border of the preserved visual field. C: Wide-field near-infrared autofluorescence identified a central area of preserved intensity indicating the area of still functioning photoreceptors. D: Wide-field optical coherence tomography shows a reduction of outer retinal layers in the center, absence of outer retinal layers in the mid-periphery and disorganization of all retinal layer towards the periphery.

Similarly, in 321/850 unsolved families (37.8 %) genetic variants could be identified that were not sufficient to explain the phenotype. This includes 86/321 families (26.8 %) with one single pathogenic or likely pathogenic variant in autosomal recessive disorders, most frequently associated the *ABCA4* gene (27/86). In the remaining 235 families, variants of uncertain significance were identified in one or more genes (Fig. 4). This underscores, that more families could be solved or likely solved with the clarification of variants of uncertain significance [12].

It has to be noted, that due to the long time period and variable testing scheme, only in a limited number of patients all currently known genes associated with IRDs have been examined. Therefore, the true number of causative variants is expected to be underestimated in this series. The huge variability of possible combinations and the uncertain relevance for the clinical course makes genotype-phenotype correlations even more futile.

## Value of genetic testing for patients

Vision is humans most valued sense [24]. In our current world visual acuity and visual fields are important for a multitude of tasks: e. g. face recognition in communication, reading, driving; it is the primary sense for most professions. Loss of visual function due to IRD is frequently associated with inability to study or work in the desired profession, a higher rate of unemployment, reduced mobility, dependence on others, loss of social status as well as fear (80 %) and depression (63 %) [25]. IRDs are the most frequent cause for acquired blindness in the working age [26,27]. For patients with childhood-onset IRDs the life-time income is reduced by one third [28]. Although a multitude of efforts have been undertaken to improve the situation of patients with visual disabilities and blindness, the progressive course of IRDs requires a process of change and acceptance [29] that can only be started when the diagnosis is confirmed.

Therefore, a fast diagnostic process is important for future life-time planning for the patients and their families. The clinical diagnosis is sufficient for initiating potential adjustments for the patients vocational training, current profession or mobility issues if required. Genetic testing is recommended to confirm the clinical diagnosis, provide genetic counselling including information concerning the individual risk of progression as well as, if required, the risk for other family members or family planning. For the identification of treatable IRDs genetic testing is mandatory, though ophthalmological treatment is currently limited to *RPE65* gene therapy and medical treatment for LHON. Especially in children genetic testing is important to differentiate between non-syndromic and syndromic IRDs. Ocular alterations might be the first sign of a syndrome with other organ manifestations accessible for treatment, genetic testing allows early detection avoiding treatment delay and reducing costs for the public health system.

While this is usually based already on the ophthalmological findings, it should not be underestimated, how important genetic testing to confirm the diagnosis is from the patient perspective [30–33]. The acceptance is high: in our center only one out of 1 433 index patients declined the initial offer of genetic testing, but decided to undergo testing 2 years later. The interest in genetic testing has several reasons: to finally confirm a clinical diagnosis and to know the origin of the individual disorder. There is the hope, that testing may find one of the few partly treatable disorders and the interest to learn about the risk for other family members, especially children. Even in devastating disorders like infantile neuroid lipofuscinosis with early death, where IRDs are one early sign, the confirmation allows the parents to understand different symptoms as one syndromic disorder, to find support in specialized clinics and to end an odyssey of repeated examinations without a final result.

It should be noted, that the availability of the first commercial gene therapy increased the acceptance of genetic testing by ophthalmologists and insurance companies. The termination of the limitation of genetic testing in the German public health system was markedly shortened the time to diagnosis and was similarly important for acceptance of genetic testing.

## Future perspectives

There are various studies related to the development of gene therapy or specific medical therapy in IRDs [34]. Regular genetic testing in all IRD patients is important for the identification of patients that can enter in such trials, as well as to determine the frequency of associated genes and even single mutations to define the resources for treatment distribution. Establishing and reminding to incorporate guidelines into clinical practice remains important.

Artificial intelligence will be helpful to identify small or subtle alterations of the retinal morphology to facilitate an earlier diagnosis of IRD patients to shorten the diagnostic odyssey and if available the time between diagnosis and treatment.

Especially for IRDs associated with syndromes, further development of networks for a patient-oriented approach of clinical care is important.
